# Structure of human V-ATPase with the *a*2-isoform reveals a proton-release gate in the luminal half-channel

**DOI:** 10.21203/rs.3.rs-11211323/v1

**Published:** 2026-10-06

**Authors:** Caitlin E. Bricault, Andrew S. Hillowe, Diksha Parwana, Ivet Bahar, Stuti Sharma

**Affiliations:** 1 Department of Biochemistry and Cell Biology, Stony Brook University, Stony Brook, NY 11794; 2 Laufer Center for Physical and Quantitative Biology, Stony Brook University, Stony Brook, NY, 11794; 3 Department of Chemistry, College of Arts and Sciences, Stony Brook University, Stony Brook, NY, 11794

## Abstract

V-ATPases are ATP-dependent proton pumps that acidify intracellular organelles in eukaryotic cells. In mammals, the V_0_ *a*-subunit has four isoforms, *a*1-*a*4, with *a*2 enriched in the Golgi and early endosomes. Mutations in *a*2 are implicated in cutis laxa, male infertility and cancer. Here, we have used cryo-EM to determine the structures of human V-ATPase with the *a*2-isoform in both the disassembled (*a*2V_0_) and assembled (*a*2V-ATPase) states. We found that *in vitro*, *a*2V-ATPase disassembles more readily than *a*4 containing V-ATPase. The structure of *a*2V_0_ allows visualization of proposed lipid binding motifs, a unique luminal extension hypothesized to sense pH in early endosomes, and the location of missense mutations that cause cutis laxa. Most strikingly, the structure of *a*2 reveals a salt bridge impeding the luminal half-channel that is conserved in vertebrates, but not in yeast. We propose that this salt bridge functions as a gating mechanism to regulate the flow of protons, and thereby V-ATPase activity in higher eukaryotes.

The vacuolar H^+^ ATPase (V-ATPase) is an ATP dependent proton pump found on the endomembrane system of virtually all eukaryotic cells, where it acidifies the lumen of intracellular organelles. Organellar acidification by the V-ATPase is required for pH and ion homeostasis, endocytosis, post-translational modifications, intracellular trafficking, neurotransmitter loading, and lysosomal degradation^1^. In specialized acid secreting cells such as osteoclasts and renal α intercalated cells, the V-ATPase localizes to the plasma membrane and acidifies the extracellular environment for bone resorption and urine acidification, respectively^2^. In addition to its canonical proton pumping function, V-ATPase participates in cellular signaling pathways such as Wnt, Notch, mTOR and AMPK^3^. Complete abrogation of V-ATPase activity is embryonic lethal in mammals, and its aberrant activity is linked to various human diseases^1,4^.

The V-ATPase is a large (~1 MDa) multisubunit complex that can be divided into a cytosolic V_1_ subcomplex and a membrane-integral V_0_ subcomplex. V_1_ is composed of subunits A-H in the stoichiometry A_3_B_3_CDE_3_FG_3_H, while V_0_ contains subunits *a*c_9_c”def along with accessory proteins ATP6AP1 and ATP6AP2. In the holo-V_1_V_0_ complex, ATP hydrolysis at the interface of the A and B subunits, drives rotation of a central rotor made of subunits D,F, d, and the c-ring (c_9_c”), along with ATP6AP1 and ATP6AP2^5–8^. Each c-subunit in the c-ring has a lipid-exposed glutamic acid which carries protons from a cytoplasmic half-channel to a luminal half-channel within the *a-*subunit, while the deprotonated glutamic acid is transiently stabilized by a conserved arginine in the *a*-subunit^9–11^. Biochemical and structural studies using the yeast V-ATPase have identified key amino acids in the *a*-subunit that constitute the proton pathway^10,12^, and more recently the role of transient water wires in assisting proton transport has been established^11^. In the absence of exogenous factors, cryo-EM of V-ATPase purified from various sources reveals three distinct rotational states of the enzyme – state 1, state 2 and state 3^5,7,8,13–15^ which are distinguished by ~120 ° rotation of the central rotor. A recent study has shown that structure-encoded soft modes of motion, uniquely defined by the overall architecture, enable the transitions between these states^16^.

V-ATPase activity is regulated by a unique process called reversible disassembly, a mechanism first described in yeast^17^ and insects^18^, but since then has been observed in mammals, including human cell lines^19–22^. In yeast, upon glucose deprivation, the V_1_ subcomplex disengages from V_0_, with concomitant silencing of V_1_-ATPase and V_0_ proton transport activities, in a process which is reversible upon glucose re-addition^23,24^. While reversible disassembly of V-ATPase is conserved across organisms, the trigger for disassembly/reassembly varies^25^. Several V-ATPase subunits can exist as tissue and/or organelle specific isoforms. Of these, the most diverse (based on number of isoforms) is the V_0_ *a*-subunit, which is central to V-ATPase’s proton pumping activity and regulation. In yeast, V_0_ *a*-subunit exists as a vacuolar (Vph1p) and Golgi (Stv1p) isoform, while in mammals the *a*-subunit has four isoforms – *a*1, *a*2, *a*3, and *a*4, which differ in tissue expression, sub-cellular localization and disease relevance. Overall, the four human *a*-subunit isoforms share 45–61% sequence identity, with *a*1 and *a*4 being most like each other. Isoforms *a*1, *a*2 and *a*3 are found ubiquitously on intercellular organelles, while *a*3 and *a*4 can be targeted to the plasma membranes of acid secreting cells^26^. Isoforms of the *a*-subunit can differ significantly in V_1_V_0_ assembly, enzyme activity and coupling efficiency, as demonstrated by studies in yeast^27^.

The *a*-subunit can be divided into a membrane integral C-terminal domain (*a*CT) and a cytoplasmic N-terminal domain (*a*NT). In yeast, the information for targeting the V-ATPase to specific subcellular compartments is contained in *a*NT^28^. Specifically, the interaction of stv1p *a*NT with PI4P has been suggested to localize stv1p containing V-ATPase to the Golgi^29,30^. In mammalian cells, the *a*2 isoform is localized to the Golgi where it plays a crucial role in glycosylation and trafficking of proteins^31^. Much like yeast, this localization has been attributed to the interaction of *a*2NT with PI4P^32^. A congenital disorder of glycosylation called cutis laxa is caused by mutations in the *a*2 isoform of V-ATPase^33–36^. In mice, in addition to cutis laxa-like symptoms, loss of function mutations in *a*2 lead to male infertility due to globozoospermia^37^. In kidney proximal tubule cells, the *a*2-isoform is found on early endosomes where it interacts with a small GTPase called Arf6 and its GEF called Cytohesin2, in a signaling role to mediate maturation of early endosomes to late endosomes by vesicular trafficking. This interaction is dependent on the luminal pH of early endosomes, and disruption of the V-ATPase-Arf6-Cytohesin2 axis inhibits endocytosis^38–40^. In ovarian tumors and cancer cell lines, increased expression of the *a*2-isoform and its localization to the cell surface have been linked to metastasis^41^. Implication of the *a*2-isoform in various diseases underscores the importance of understanding its unique properties and molecular structure.

In this study, we have used cryo-EM to determine the structures of human V-ATPase containing the *a*2 isoform, as the assembled holo-enzyme (*a*2V-ATPase), and the disassembled, autoinhibited subcomplex (*a*2V_0_). Comparison of *a*2V-ATPase and *a*4V-ATPase reveal that *a*2V-ATPase is more prone to disassembly, hinting at their differential susceptibilities for regulated disassembly *in vivo*. The high-resolution structure of *a*2V_0_ reveals proposed PI4P lipid binding motifs^32^, and a unique luminal extension that has been hypothesized to sense pH in early endosomes^38^. The structure also allows mapping of missense mutations found in patients with cutis laxa, with several mutations located along the proton pathway. In the *a*2V_0_ structure, we found that the access of the luminal half-channel to polar residues important for proton transport is interrupted by a salt bridge, which is conserved in vertebrates, including human *a*-isoforms, but absent in yeast. MD simulation reveals that this salt bridge is transient and expected to break in alternative conformations of the enzyme. Based on our findings, we propose that this salt bridge acts as a gate to control the flow of protons, and in turn regulate V-ATPase activity in higher eukaryotes.

## Results

### Overall structure of human *a*2V0 and *a*2V-ATPase.

A HEK293F suspension cell line expressing C-terminally FLAG-tagged *a*2-isoform was a kind gift from Dr. Rebecca Oot (SUNY Upstate Medical University). Immunofluorescence of the stable cell line suggested localization of *a*2-FLAG to the ER, trans-Golgi network, Rab5- and Rab7- positive endosomes, and Lamp1 positive endo-lysosomes (Extended data fig. 1A). Following a published method^42^, *a*2V-ATPase was extracted from total cell membranes using lauryl maltose neopentyl glycol and cholesteryl hemisuccinate (LMNG-CHS), purified using anti-FLAG affinity chromatography and subjected to glycerol gradient centrifugation for separation of free *a*2V_0_ (Fig. 1A) from intact *a*2V-ATPase. Mass spectrometry of excised bands from SDS-PAGE of purified *a*2V_0_ helped confirm its components, and identify contaminants like PRMT5 and WDR77, which are commonly found in FLAG-affinity purified proteins from HEK293 cells (Extended data fig. 1B)^43^. Purified *a*2V_0_ was subjected to single particle cryo-EM to determine a map at ~3.2 Å resolution (Fig. 1B, Extended data fig. 2). The map allowed building of an atomic model (Fig. 1C) using an Alphafold-3^44^ predicted model of the human *a*2-isoform and structure of the human *a*1V-ATPase (PDB: 6WM4)^5^. Consistent with previous structures of V_0_^9–11^, *a*2V_0_ occupies rotational state 3 in its autoinhibited conformation. Topologically, subunit-*a*2 can be divided into a cytosolic *a*2NT (amino acids 1–372) composed of nine α-helices (NH1-NH9) and nine β-strands; and a membrane integral *a*2CT composed of fourteen α-helices (CH1-CH14) (Extended data fig. 1C). Amino acids 2–845 have been resolved in our structure, except for a disordered region in *a*2NT (residues 136–174) and another region in *a*2CT (residues 678–725), consistent with previous work^9–11^.

Purified *a*2V-ATPase (Fig. 1D) was subjected to single particle cryo-EM resulting in three maps at ~3.6 Å, ~4.3 Å and ~3.8 Å resolution, corresponding to rotational states 1, 2 and 3, respectively. The resolution of different regions of the map was improved using focused refinement, resulting in composite maps that allowed the building of atomic models for states 1 and 3 (Fig. 1E,F, Extended data fig. 3 and 4). The cryo-EM density for subunit-H could not be improved by focused refinement, and so it is not included in the models for States 1 and 3, similar to previous work^7^. Surprisingly, unlike published studies^5,13,15,45^, where state 3 is typically the least abundant, state 2 was the least abundant and poorly resolved in our study (Fig. 1E). In addition, when compared to the *a*1V-ATPase state 1 structure (PDB: 6WM2)^5^, the position of the *a*2V-ATPase state 1 c-ring is ~18° rotated in the counterclockwise direction with respect to *a*2 (when observed from the cytosol towards the membrane) (Fig. 1G), with no significant structural changes in V_1_. A similar ~14° rotated conformation has been observed in autoinhibited yeast V_0_, suggesting that minor rotation in the V-ATPase c-ring can occur without ATP hydrolysis^11^. At this position, the presumably deprotonated cE139 is stabilized by *a*2H816 and cY68 (Fig. 1H). In yeast, point mutation of Vph1H796, which corresponds to *a*2H816, leads to almost complete loss of V-ATPase activity and no growth complementation at pH 7.5, highlighting its importance in proton transport^12^

### Stability of V-ATPase varies between the human *a*2 and *a*4 isoforms.

As previously reported for *a*4V-ATPase in detergent^42^, purified *a*2V-ATPase in LMNG-CHS did not exhibit significant ATPase activity. When reconstituted into lipid nanodiscs (Extended data fig. 5A-C), *a*2V-ATPase showed concanamycin A sensitive V-ATPase activity, lower than, but comparable to *a*4V-ATPase, both their activities being significantly lower than the yeast V-ATPase (Extended data fig. 5D). Of note, purified *a*4V-ATPase and *a*2V-ATPase in lipid nanodiscs were not as homogeneous as the yeast V-ATPase in lipid nanodiscs (Extended data fig. 5E-G). To ensure accurate ATPase activity measurements, the concentrations of *a*2V-ATPase and *a*4V-ATPase in lipid nanodiscs were calculated using SDS-PAGE of known amounts of the yeast enzyme as standard. While specific activities of *a*2 and *a*4 containing V-ATPase were somewhat similar, their relative assembly differed significantly. *a*4V-ATPase and *a*2V-ATPase were purified in LMNG-CHS using the same procedure, and equal amounts of purified protein were subjected to glycerol density gradient centrifugation. We found that while significant amounts of *a*4V-ATPase migrated to denser fractions as intact V-ATPase, *a*2V-ATPase mostly remained in lighter fractions as free *a*2V_0_ (Fig. 2A–C). Furthermore, the fractions that appeared to contain intact *a*2V-ATPase, showed mostly disassembled subcomplexes upon negative stain electron microscopy. Compared to *a*4V-ATPase, ~50% of *a*2V-ATPase showed assembled V_1_V_0_ (Fig. 2D–F), suggesting that *a*2V-ATPase is inherently more labile compared to *a*4V-ATPase, at least in detergent micelles. Studies using yeast V-ATPase have shown that V_1_ and V_0_ are held together by low affinity but high avidity interactions, and central to these interactions is the cytosolic *a*NT domain^46,47^. Our observation of two different *a*-subunit isoforms affecting the stability of V_1_ and V_0_ is reminiscent of yeast studies that show Vph1p containing V-ATPase undergoes obvious glucose-dependent reversible disassembly but the Stv1p-containing V-ATPase does not^27^.

### Unique structural features of the *a*2-isoform.

The structure of *a*2V_0_ shows a well-ordered loop that extends out of the detergent micelle, into what would be the lumen of an organelle (Fig. 3A,B, *Extended data movie 1*). On either side of the loop, we observe density extending from *a*2N484 and *a*2N505 consistent with glycosylation, which has biochemically been demonstrated for these residues^48^. We have therefore modelled N-acetyl glucosamine (NAG) into these densities (Fig. 3B). Alignment of human *a*1 and *a*4 isoforms with our *a*2 structure shows that while the *a*1 and *a*4 glycosylation sites are in roughly the same position, the two glycosylation sites in *a*2 are positioned on either side of the *a*1 and *a*4 NAG sites (Fig. 3C). In *a*2, the position between the two glycosylation sites is occupied by the luminal loop discussed above (Fig. 3B,C). Sequence alignment shows that of all the human *a*-subunit isoforms, this luminal extension is only found in *a*2 (Fig. 3E). This loop, and the succeeding C-terminal helix, house a cluster of three histidines (H493, H498 and H511) (Fig. 3D), two of which are conserved among *a*2-subunits in mammals (Extended data fig. 7). Interestingly, these luminal histidines are exclusively found in the *a*2-isoform (Fig. 3E). *Marshansky and colleagues* have hypothesized that these histidines act as luminal pH sensors, that can influence the conformation of *a*2 to promote interaction of V-ATPase with Cytohesin-2, a phenomenon which is required for vesicular trafficking from early endosomes to late endosomes in kidney proximal tubule cells^38,40,49^.

### Lipid interactions.

In the center of the c-ring, we observed density corresponding to ordered lipids as previously reported for both human and yeast structures^5,10,11^. The density aligned well with *a*1V-ATPase structures^5^, and so were modeled as phosphatidyl choline and cholesterol (Extended data fig. 4A). PI4P is enriched in Golgi and has been proposed to localize *a*2V-ATPase to the Golgi by directly interacting with *a*2NT^30^. Purified *a*2NT in solution binds to PI4P via two possible motifs – K^221^WY and GK^184^V, with mutation of the former inhibiting direct interactions^32^. Both these motifs have been resolved in our *a*2V_0_ structure. The K^221^WY motif sits on a β-hairpin loop (Fig. 3A,F), while the GK^184^V motif sits on a membrane adjacent helix (NH5) (Fig. 3A,G), both located in the distal lobe of *a*2NT. Cryo-EM density for *a*2K221 and *a*2K184 face away from the membrane in our structure (Fig. 3H,I). However, the proximity of this region to the membrane suggests that a conformational change could promote *a*2NT’s interaction with PI4P.

### The proton pathway.

Based on a comparison of our *a*2V_0_ structure with structural and biochemical experiments conducted in yeast^9–12^, we have highlighted key amino acids involved in proton translocation in human *a*2V-ATPase (Fig. 3J–M). Interfacing with the c-ring are two horizontal helices of *a*2, which correspond to CH13 and CH14 (Fig. 3J, Extended Fig. 1C). Roughly in the center of the horizontal helices lies a positively charged patch composed of R819, H816 and R755, with R755 forming a salt bridge with the deprotonated c”E98 (Fig. 3J,L). On either side of this positively charged patch are two solvent exposed cavities (Fig. 3J). The cytoplasmic cavity has a calculated volume of ~3284 Å^3^ and lies at the interface of *a*2CT (helices CH8, CH13 and CH14) and the c”-subunit, and is lined by polar residues – N827, N749, S748, E741 and H738 (Fig. 3J,M). In the *a*2V_0_ structure, the calculated volume of the luminal cavity is ~1733 Å^3^, which is significantly smaller than that in the yeast V_0_ structure (PDB: 6M0R) (~3884 Å^3^)^11^. Based on previous studies in yeast^11,12^, polar residues that outline the luminal cavity in *a*2V_0_ are E809, H763, H417 and D414. However, in our *a*2V_0_ structure, this cluster of polar residues does not directly surround the luminal cavity (Fig. 3J,K). The importance of this discrepancy is discussed in the subsequent section. Consistent with the proposed role of *a*2E809 (E798 in yeast Vph1p) in the transfer of protons from cE139 via a cluster of transient water molecules^11^, in our structure of *a*2V_0_, *a*2E809 sits directly opposite cE139 (Fig. 3L). On either side of the salt bridge between *a*2R755 and c”E98, are additional polar residues (H816, S812 and S752) (Fig, 3L), that probably stabilize the deprotonated c-ring glutamate, while it makes its way from the luminal cavity to the cytoplasmic cavity.

### A salt-bridge gate is a putative regulatory mechanism in human V-ATPase.

While calculating the luminal cavity in the *a*2V_0_ structure, we made a surprising observation. In the yeast V_0_ structure, the luminal cavity extended up to the cluster of polar residues that are implicated in proton transfer (Fig. 4A,C,D), but in the human *a*2V_0_ structure, the luminal cavity falls short (Fig. 4A,B,E). In *a*2V_0_, the luminal cavity is impeded by a salt bridge between R543 and D768 (Fig. 4E,F,G, *Extended data movie 2*). Interestingly, while R543 and D768 are highly conserved between the *a*2 sequences of vertebrates (Extended data fig. 7), in yeast these positions are occupied by alanine, glycine and serine (Fig. 4H). We replaced R543 to A and D768 to S in our *a*2V_0_ model *in silico* and recalculated the luminal cavity, which now extended all the way to the cluster of polar residues, much like in yeast V_0_ (Fig. 4I), suggesting that the R543-D768 salt bridge in *a*2V_0_ is impeding access of the luminal cavity. To assess whether this impediment is exclusively a feature of autoinhibited *a*2V_0_, we calculated the luminal cavity in our structures of *a*2V-ATPase in states 1 and 3. A similar premature obstruction of the luminal cavity was found in intact *a*2V-ATPase (Extended data fig. 6A,B). In other human *a*-subunit isoforms, a positively charged amino acid is found in place of R543 and a negatively charged amino acid is found in place of D768 (Fig. 4H). When we calculated luminal cavities using existing structures of *a*1V-ATPase (PDB: 6WM2)^5^ and *a*4V-ATPase (PDB: 7UNF)^6^, we found a similar interruption of the luminal cavity (Extended data fig. 6C,D), suggesting that this is a conserved phenomenon in human V-ATPase *a*-isoforms.

Based on our structural data, we hypothesize that the R543-D768 salt bridge acts as a “gate” to modulate proton flow and thereby regulate V-ATPase activity. However, to function as a gate, the R543-D768 salt bridge must be transient, such that it is broken when the enzyme is actively pumping protons. To test this, we conducted molecular dynamics (MD) simulation using the structure of *a*2V_0_. During three independent 100 ns simulations, R543 and D768 remained within salt bridging distance less than ~40% of the time, suggesting that the *a*2R543-D768 salt bridge observed in our structure is transient in other enzyme conformations (Fig. 4J,M, *Extended data movie 3*). In our simulations, R543, when not in contact with D768, was often stabilized by D470 (Fig. 4K–M). We calculated the luminal cavity for this potentially alternate conformation with an R543-D470 salt bridge, and found the luminal cavity extended all the way to E809, the residue involved in proton uptake from cE139 (Fig. 4N). Of note, even though R543 and D768 are not conserved between yeast and humans, D470 is conserved, and mutation of the corresponding residue (D481) in yeast leads to significant reduction in proton transport activity, and inability to complement the growth phenotype at pH 7.5, suggesting its importance in the proton transport mechanism^12^.

### Missense mutations found in patients with Cutis Laxa.

Cutis laxa is a congenital disorder of glycosylation manifested in redundant, inelastic skin along with growth and developmental defects. Several mutations in ATP6V0A2, the gene that encodes the *a*2-isoform, have been reported in patients with cutis laxa. Observed phenotypes are due to impaired function of *a*2V-ATPase in the Golgi leading to glycosylation defects, abnormal trafficking of proteins through the Golgi, and aberrant secretion^34^. Most of the mutations reported in ATP6V0A2 are predicted to be loss of function due to improper splicing, frameshift, deletions or premature stop codons. However, several missense mutations have been reported, for which the causation of disease is not as straightforward^50^. We have mapped the position of missense mutations in the structure of *a*2V_0_ to speculate on the underlying reason for disease. The reported missense mutations can be broadly divided into proline-related mutations characterized by a mutation of a proline, or to proline residue (Fig. 5A–F), mutations in the proton pathway (Fig. 5B,G,H), and a single mutation in the luminal extension (Fig. 5A,I). Proline-related mutations are known to alter secondary structure characteristics, including in transmembrane regions of proteins, which can lead to protein instability^51^. This phenomenon has been experimentally demonstrated for the cutis laxa causing P405L mutation^52^ as discussed below. The P87L mutation is compound heterozygous with a frameshift mutation in the other copy, manifested in a mild cutis laxa phenotype^33^. P87 is located in the loop connecting NH3 and NH4 in *a*2NT and presumably contributes to the lack of secondary structure in this region (Fig. 5A,C). This residue is not conserved in vertebrates and is flanked by multiple other prolines, that can offer redundancy to its role (Extended data fig. 7). A cutis laxa patient with moderate symptoms was homozygous for two missense mutations – P405L and R510I^33^. P405 lies in the second C-terminal helix (CH2) of *a*2CT, while R510 lies in the luminal extension of *a*2CT (Fig. 5A,D,I). Studies conducted in HEK293T cells suggest that P405 is important for proper folding and processing of *a*2^52^. R510I, located in the luminal extension of *a*2CT, is near a glycosylation site on subunit-e and RNaseK/f-subunit’s E87 (Fig. 5I). However, the side chain density for R510 is poorly resolved, and this residue is not conserved in vertebrates (Fig. 5I, Extended data fig. 7), making it unlikely that R510 is involved in interactions that are required for the stability of *a*2V_0_. Therefore, the evidence thus far suggests that P405L is the disease-causing mutation in this patient. *Huchtagowder et al*. reported two different patients harboring the P792R mutation with severe phenotypes^33^. P792 is located on one of the two horizontal helices (CH14) which participate in proton transport (Fig. 5B,E). The residue does not directly participate in proton transport but is potentially required for stability of the secondary structure in the region. A similar mutation found on CH14 is L811P (Fig. 5B,F)^35^, which potentially has similar effects as the P792L mutation. Interestingly, three of the four mutations found along the proton pathway are in the region that surrounds the luminal cavity (Fig. 5B,G). The three mutations G416R, H417Y and H763Y were reported in three different patients, who were compound heterozygous and showed severe phenotypes^35,53^. Their location on our structure indicates potential dysregulation of proton pumping activity in these patients. Lastly, a patient homozygous for the mutation S752F showed severe phenotype^54^. S752 is located along the proton pathway between R755 and the cytoplasmic cavity, indicating that S752 might help stabilize a deprotonated c-ring glutamate while it traverses between luminal and cytoplasmic cavities (Fig. 5B,H).

## Discussion

Our study has resolved the cryo-EM structures of human *a*2V-ATPase and *a*2V_0_. In the process, we discovered that *a*2V-ATPase is more labile compared to *a*4V-ATPase, existing mostly as disassembled *a*2V_0_ *in vitro*. Our finding is consistent with a published study that purified predominantly disassembled *a*2V_0_ from bovine lungs using conventional purification methods, without affinity chromatography^55^. While *a*2 is typically found on moderately acidic compartments such as Golgi (pH 6.0 to 6.7)^56^ and early endosomes (pH 6.0 to 6.5)^57^, *a*4V-ATPase in renal cells is designed to acidify the large extracellular environment^26^. To regulate V-ATPase activity on Golgi, and to maintain optimal pH for glycosylation, two proteins – Oxr1 and Ncoa7 disassemble the V-ATPase *in vivo*^58^. Studies conducted in yeast show that in response to glucose removal, Oxr1 interacts with V-ATPase in state 1 to efficiently disassemble the enzyme into V_1_ and V_0_^45^. On the other hand, it has been suggested that since autoinhibited V_0_ occupies state 3, and in most of the available V-ATPase structures state 3 is the least populated, high energy conformation, disassembly of V-ATPase occurs in rotational state 3^15,59^. In our *a*2V-ATPase cryo-EM dataset, the number of particles in state 3 is enriched compared to state 2 (Fig. 1E), and our biochemical data shows that that purified *a*2V-ATPase is easily disassembled (Fig. 2). Taken together, we speculate that *a*2V-ATPase is designed for easy disassembly, befitting its localization to moderately acidified compartments.

One of our most surprising findings was the existence of a salt bridge gate that limits access of the luminal cavity, potentially regulating proton transport. Impediment to the flow of protons by this salt bridge might explain why human *a*2V-ATPase and *a*4V-ATPase show a reduction in coupled ATPase activity when compared to yeast V-ATPase (Extended data fig. 5D). Furthermore, single molecule experiments conducted using rat brain synaptic vesicles demonstrated a subset of assembled V-ATPases that were inactive, even in the presence of ATP^60^. Additional regulatory mechanisms in mammalian V-ATPase might explain the observations described above, and we think the *a*2R543-D768 salt bridge gate is a contender for such a role. A salt bridge blocking the luminal cavity has been observed in all other available human *a*-isoform structures (Extended data fig. 6). Whether the salt bridge is also present in the *a*3-isoform is currently unknown as there is no structure of *a*3 available. Interestingly, at the same position as *a*2R543, *a*3 has a histidine (*a*3H526) (Fig. 4H), which typically has a pK_a_ in the physiological range and its protonation/deprotonation can dictate salt bridge formation, and consequently access of the luminal cavity. Along the same lines, protonation of *a*2D768 can also destabilize the *a*2R543-D768 salt bridge. The pK_a_ of *a*2D768 in our structure is ~4.5, as predicted by PROPKA^61^, so we expect *a*2D768 to be deprotonated under most physiological circumstances. However, even when the charge on *a*2D768 is neutralized, the positive charge on a *a*2R543 would repel proton transport, unless it was stabilized by another residue such as *a*2D470. The predicted pK_a_ of *a*2D470 based on our structure is ~8.45, consistent with it being buried in a hydrophobic environment, but this would most likely change when the luminal cavity is open and *a*2D470 is exposed to an aqueous environment. Extensive mutagenesis along with proton transport assays and structures of mutants are required to delineate the mechanism of how the *a*2R543-D768 salt bridge regulates V-ATPase activity.

Lastly, our structures directly inform on the position of missense mutations in the *a*2-isoform that cause cutis laxa. Several of these are proline related mutations, which presumably affect the stability of the protein, but their effect on other aspects of V-ATPase function cannot be discounted. Mutations in the proton pathway could result in either reduction or increase in proton pumping activity, and in some cases, V-ATPase assembly or coupling between V_1_ and V_0_ might also be affected. Therefore, further investigation is required to understand the mechanism behind missense mutations that cause cutis laxa.

## Methods

### Cell culture.

HEK293F suspension cell lines expressing C-terminally 2xFLAG-tagged *a*2-isoform and *a*4-isoform were a kind gift from Dr. Rebecca Oot (SUNY Upstate Medical University)^42^. Cells were cultured in SMM 293-TII serum free culture medium (Sino Biological US Inc.) and 100 μg/ml Geneticin (Gibco) at 37 °C, in a humidified 8% CO_2_ atmosphere. Cells from 1.6 L cultures were harvested at a density of ~3 ×10^6^ cells/ml by centrifugation and flash frozen until further use. A yeast strain deleted for the VMA10 gene (encoding G-subunit) SF838–5Aα vma10Δ::KanMX and a pRS315 plasmid encoding the G-subunit with an N-terminal FLAG-tag was a kind gift from Dr. Stephan Wilkens (SUNY Upstate Medical University)^62,63^. Yeast strain SF838–5Aα vma10Δ transformed with pRS315 encoding FLAG tagged G-subunit was grown to late log phase in YPD pH 5 media and harvested by centrifugation.

### Purification of human V-ATPase in detergent.

Human V-ATPase containing the *a*2-isoform, and the *a*4-isoform were purified according to previously published methods^42^, with some modifications. Briefly, cell pellets were resuspended in equal volume of 25 mM Tris pH 7.5, 150 mM NaCl, 0.5 mM EDTA (TBSE) supplemented with protease inhibitors. Cells were lysed using Dounce homogenization and membranes collected by centrifugation at 15,000 xg for 1 hour at 4 °C. The membrane pellet was resuspended in equal volume of TBSE and diluted to ~10 mg/ml membrane protein concentration. Membranes were solubilized by adding 1% LMNG and 0.1% CHS and incubated at 4 °C for 4 hours. Solubilized membranes were cleared by centrifugation at 16,000 xg for 15 mins and the supernatant was applied to a pre-equilibrated FLAG column, washed with 20 column volumes of TBSE with 0.02% LMNG, 0.002% CHS and eluted using the same buffer supplemented with 0.25 mg/ml of 3x FLAG peptide (APExBIO). Eluted fractions were analyzed by SDS-PAGE and relevant fractions were concentrated and layered on a 10–40% glycerol gradient. After centrifugation at 180,000 xg for 14 hours at 4 °C, the 11 ml gradient volume was fractionated into 18 × 600 μl fractions. The fractions were analyzed by SDS-PAGE and relevant fractions were pooled and concentrated using an Amicon Ultra 100 kDa MWCO centrifugal concentrator (Millipore Sigma).

### Purification of human V-ATPase in lipid nanodiscs.

Nanodisc reconstitution of human *a*2V-ATPase and *a*4V-ATPase was conducted according to published methods^42^. Briefly, membrane pellets were resuspended in 1% (w/v) n-dodecyl-β-D-maltoside (DDM) (Anatrace) and membrane scaffold protein (MSP1E3D1 – purified as described in^42^) added at a molar ratio of 1:2.5 membrane protein: MSP, in presence of protease inhibitors. The mixture was incubated for 45 minutes at 4 °C and centrifuged at 16,000 xg for 15 minutes to remove insoluble material. From the supernatant, detergent was removed using 0.4 g/ml of Bio-beads SM2 (Bio-Rad) for 2 hours at 4 °C, which allowed self-assembly of membrane proteins into lipid nanodiscs. From a heterogenous mixture of membrane proteins in nanodiscs, V-ATPase containing nanodiscs were purified using FLAG affinity chromatography followed by glycerol density gradient centrifugation as described above. The fractions were analyzed by SDS-PAGE and relevant fractions were pooled and concentrated using an Amicon Ultra 100 kDa MWCO centrifugal concentrator (Millipore Sigma).

### Purification of yeast V-ATPase in lipid nanodiscs.

Yeast V-ATPase was purified and reconstituted into lipid nanodiscs as described previously^62^. Briefly, SF838–5Aα vma10Δ transformed with pRS315 encoding FLAG tagged G-subunit grown to late log phase was harvested and used for vacuole isolation as described previously^62^. Vacuoles supplemented with protease inhibitors were detergent solubilized using 1.2 mg of DDM per 1 mg of vacuolar membrane protein for 1 hour at 4 °C. Insoluble material was pelleted by centrifugation at 100,000 xg for 15 minutes at 4 °C. MSP was added to the supernatant at a molar ratio of 1:50 and incubated for 1 hour at 4 °C, followed by detergent removal using Bio-beads SM2 at 0.6 g/ml for 2 hours at 4 °C. V-ATPase in lipid nanodiscs was then purified using FLAG affinity chromatography, elution fractions were analyzed by SDS-PAGE and relevant fractions were pooled and concentrated using an Amicon Ultra 100 kDa MWCO centrifugal concentrator (Millipore Sigma).

### Cryo-EM sample preparation and data collection.

3 μl *a*2V_0_ at 4 mg/ml in TBSE and 3 mM fluorinated Fos-choline-8 (Anatrace) was applied to glow discharged UltrAuFoil R1.2/1.3 grids (Quantifoil) and blotted for 4.5 s with 100% humidity before plunge freezing in liquid ethane using a Vitrobot Mark IV system (Thermo Fisher Scientific). Cryo-EM images were collected on a Talos Arctica operated at 200 kV, equipped with a Falcon 4i detector (Thermo Fisher Scientific) at the Stony Brook University Cryo-EM facility. Movies were recorded as .EER files at a magnification of x92,000, corresponding to a pixel size of 1.1 Å, with a total dose of 40 e/Å^2^, at a defocus range of −0.8 to −1.5 μm.

To collect the cryo-EM dataset of *a*2V-ATPase, 3 μl of the purified complex at 1 mg/ml was applied to glow discharged UltrAuFoil R1.2/1.3 grids, incubated for 10 minutes followed by manual blotting and another 3 μl of sample was applied and blotted for 4.5 s with 100% humidity before plunge freezing in liquid ethane using a Vitrobot Mark IV system (Thermo Fisher Scientific). Cryo-EM images were collected using a Talos Arctica operated at 200 kV, equipped with a Falcon 4i detector (Thermo Fisher Scientific) at the Stony Brook University Cryo-EM facility. Movies were recorded as .EER files at a magnification of x92,000, corresponding to a pixel size of 1.1 Å, with a total dose of 40 e/Å^2^, at a defocus range of −0.8 to −1.4 μm.

### Cryo-EM image processing.

All cryo-EM image processing steps were conducted in CryoSPARC v4.6.2^64^. Raw .EER movies were corrected for beam-induced motion and contrast transfer function using patch motion correction and Patch CTF. For *a*2V_0_, a template was generated using manual picker. Template picker was then used to pick particles from the dataset. Extracted particles were subjected to multiple rounds of 2D classification followed by *Ab inito* reconstruction, heterogenous refinement, homogenous refinement, 3D classification and local refinement. The final resolution of the map was determined using Fourier shell correlation with the gold-standard cutoff of 0.143 (Extended data fig 2). For *a*2V-ATPase, after Patch motion correction and Patch CTF, manual picker, template picker and Topaz^65^ were used to pick and extract particle images. Multiple rounds of 2D classification were followed by *Ab initio* reconstruction, heterogenous refinement and homogenous refinement to generate three consensus maps for States 1, 2 and 3. For each conformation, signal subtraction and focused refinement was used to improve the resolution of specific regions. The final resolution of each map was determined using Fourier shell correlation with the gold-standard cutoff of 0.143 (Extended data fig 3).

### Model building and refinement.

Subunits *c, c”, d1*, ATP6AP1 and ATP6AP2 were rigid body fit from the structure of *a*1V-ATPase (PDB: 6WM4)^5^ into their corresponding cryo-EM density in the *a*2V_0_ map, while the Alphafold-3^44^ predicted model of the human *a*2-isoform was fit into the density corresponding to *a*2. The model was adjusted first in coot v0.9.1^66^ followed by ISOLDE^67^ and finally real space refined in PHENIX v2.0^68^. The model was validated using MolProbity^69^, as implemented in PHENIX. Maps and models were visualized, and figures were generated using UCSF ChimeraX v1.11.1^70^. For *a*2V-ATPase structures in States 1 and 3, the *a*2-subunit from our *a*2V_0_ model was used as a reference, and for the rest of V-ATPase subunits the structures of *a*1V-ATPase (PDB: 6WM2 for State 1 and 6WM4 for State 3)^5^ were used as references. Each model was adjusted using coot and ISOLDE against the focused map corresponding to V_0_, V_1_ or the peripheral stator (Extended data Fig 3E). The adjusted model was refined against the consensus map in PHENIX and validated using MolProbity, as implemented in PHENIX.

### Calculation of cavities.

For measurement of luminal and cytosolic cavities, KVFinder-web^71^ was used with the following parameters – probe in 1.4 Å, probe out 6 Å, removal distance 3 Å and volume cutoff 150 Å^3^.

### Molecular dynamics simulations.

The *a*2V_0_ structure, comprising subunit-*a*2, the c-ring, subunits d1, e1, and f/RNaseK, along with the accessory subunits ATP6AP1/Ac45 and ATP6AP2/PRR, was embedded in a POPC bilayer and solvated using CHARMM-GUI Membrane Builder^72^. All 16 chains resolved in the experimental structure were retained. The resulting system contained 302 POPC molecules, ~70,000 TIP3P water molecules, and Na^+^/Cl^−^ ions at 0.15 M, including those required for charge neutrality, for a total of ~298,000 atoms in a box of approximately 130 × 130 × 187 Å^3^. Protein, lipids, and ions were described by the CHARMM36m/CHARMM36 force fields together with the TIP3P water model. All simulations were performed with NAMD 3.0.1^73^. After energy minimization, the system was equilibrated for ~2 ns using the standard six-stage CHARMM-GUI protocol, in which positional restraints on protein heavy atoms and restraints on lipid headgroups and tails were gradually released. Production simulations were then run without restraints in the NPT ensemble at 310 K and 1 atm, maintained by Langevin dynamics (damping coefficient 1 ps^−1^) and a Nosé–Hoover Langevin piston, respectively^74^. Three independent replicas of 100 ns each were run, and coordinates were saved every 100 ps for analysis. Salt-bridge formation was analyzed using MDTraj^75^. For each frame, the interaction was described by the minimum distance between the three guanidinium nitrogen atoms of Arg543 (NE, NH1, NH2) and the two carboxylate oxygen atoms of the partner aspartate (OD1, OD2); a salt bridge was considered formed below a 4.5 Å cutoff. Distance traces are shown as a 1.5 ns rolling mean over a common 100 ns window.

### Assessment of stability between *a*2V-ATPase and *a*4V-ATPase.

Following affinity purification, elution fractions containing either *a*4V-ATPase or *a*2V-ATPase were concentrated separately. Equal amounts of *a*4V-ATPase or *a*2V-ATPase were applied on top of 10% to 40% glycerol gradients and centrifuged at 180,000 xg for 14 hours at 4 °C. The 11 ml gradients were fractionated into 600 μl fractions and analyzed by SDS-PAGE. For quantification of ATP6V1A bands, the gels were stained with SYPRO Ruby protein gel stain (ThermoFisher) and analyzed by densitometry using ImageJ^76^.

### Negative stain EM image collection and analysis.

For *a*4V-ATPase and *a*2V-ATPase, glycerol gradient fractions consistent with V_1_ and V_0_ subunit bands were analyzed using negative stain electron microscopy. 3 μl of sample was applied to glow-discharged 150-mesh carbon-coated copper grids (Electron Microscopy Sciences) and stained with 1.5% (w/v) uranyl formate solution (Electron Microscopy Sciences). Images were captured using an FEI BioTwin G2 transmission electron microscope operating at 80kV at a nominal magnification of 120,000x with an AMT X60 CCD camera. Image processing was performed using Simplified Application Managing Utilities for EM Labs (SAMUEL v.21.01)^77^. Assembled and disassembled V-ATPase particle images were selected using template-based picking in samautopick.py, and 2D classification was conducted using samclasscas.py. 20 images were analyzed per purification, for three separate purifications. The number of assembled V-ATPases per micrograph was assessed by 2D classification and the statistical significance between groups was determined using an unpaired, two-tailed Welch’s t-test.

### Immunofluorescence and confocal microscopy.

HEK293F cells stably expressing the *a*2-isoform at a cell density of 0.25 × 10^6^ cells/ml were plated on 18 mm glass coverslips coated with 0.01% L-polylysine (Millapore Sigma) and incubated for 30 minutes. Coverslips were washed with phosphate buffer saline (PBS), then fixed with 4% paraformaldehyde (Electron Microscopy Sciences) for 25 minutes at room temperature. Cells were then permeabilized with PBS containing 0.1% Triton X-100 at room temperature for ~7 minutes. Coverslips were washed with PBS and blocked with 5% bovine serum albumin for ~1 hour at room temperature. Coverslips with immobilized cells were then incubated with a 1:200 dilution of anti-FLAG (Proteintech) and anti-calnexin (ThermoFisher), anti-TGN46 (Proteintech), anti-Rab5 (ThermoFisher), anti-Rab7 (Cell Signaling), or anti-LAMP1 (Cell Signaling) primary antibodies ~1 hour at room temperature. Cells were washed 3 × 10 minutes with PBS then immunostained with Alexa Fluor 488 goat anti-rabbit IgG (Thermo Fisher Scientific) for organelles and Alexa Fluor 555 goat anti-mouse IgG (Thermo Fisher Scientific) for *a*2-FLAG. Each secondary antibody was diluted 1:500 and incubated at room tempaerature for 1 hour along with 5 μg/ml of 4′,6-diamidino-2-phenylindole (DAPI) (Millipore Sigma). Following three PBS washes, coverslips were mounted with ProLong Gold Antifade Reagent (Fisher Scientific). Immunolabeled samples were imaged on a Zeiss Laser Scanning Mircoscope 980 Non-Linear Optics confocal system with an inverted Axio Observer 7 stand. Excitation was performed using 405, 488, and 561 nm lasers, with signal detection configured across two Multi-Alkali Photomultiplier Tube detectors and one Gallium Arsenide Phosphide detector using a 63×/1.40 Numerical Aperture Oil objective. Image compositions were acquired using Zeiss 3.6 (Blue edition) and post-processing performed with ZEN 3.12 software.

### ATPase activity measurements.

MgATPase activity of purified human and yeast V-ATPase was measured using a coupled enzyme assay as described previously^62^. Each reaction contained 25 mM HEPES pH 7.5, 4 mM MgCl_2_, 1 mM NADH, 7.5 mM phosphoenolpyruvate, 2.5 mM ATP, 18.8 U/ml pyruvate kinase, and 21.5 U/ml lactate dehydrogenase. Assays were performed at 37 °C in a 384-well microplate in a total reaction volume of 30 μl. Due to some heterogeneity in the human V-ATPase nanodisc preparations, yeast V-ATPase in nanodiscs was used as a standard to determine protein concentration by SDS-PAGE of increasing concentrations of protein. The rate of decrease in absorbance at 340 nm was converted to the rate of ATP hydrolysis using the molar extinction coefficient of NADH (6,220 M^−1^ cm^−1^). To assess coupled V-ATPase activity, concanamycin A (Santa Cruz Biotechnology) was added to the assay at 0.2 μM final concentration.

## Extended Data

**Extended data figure 1.**
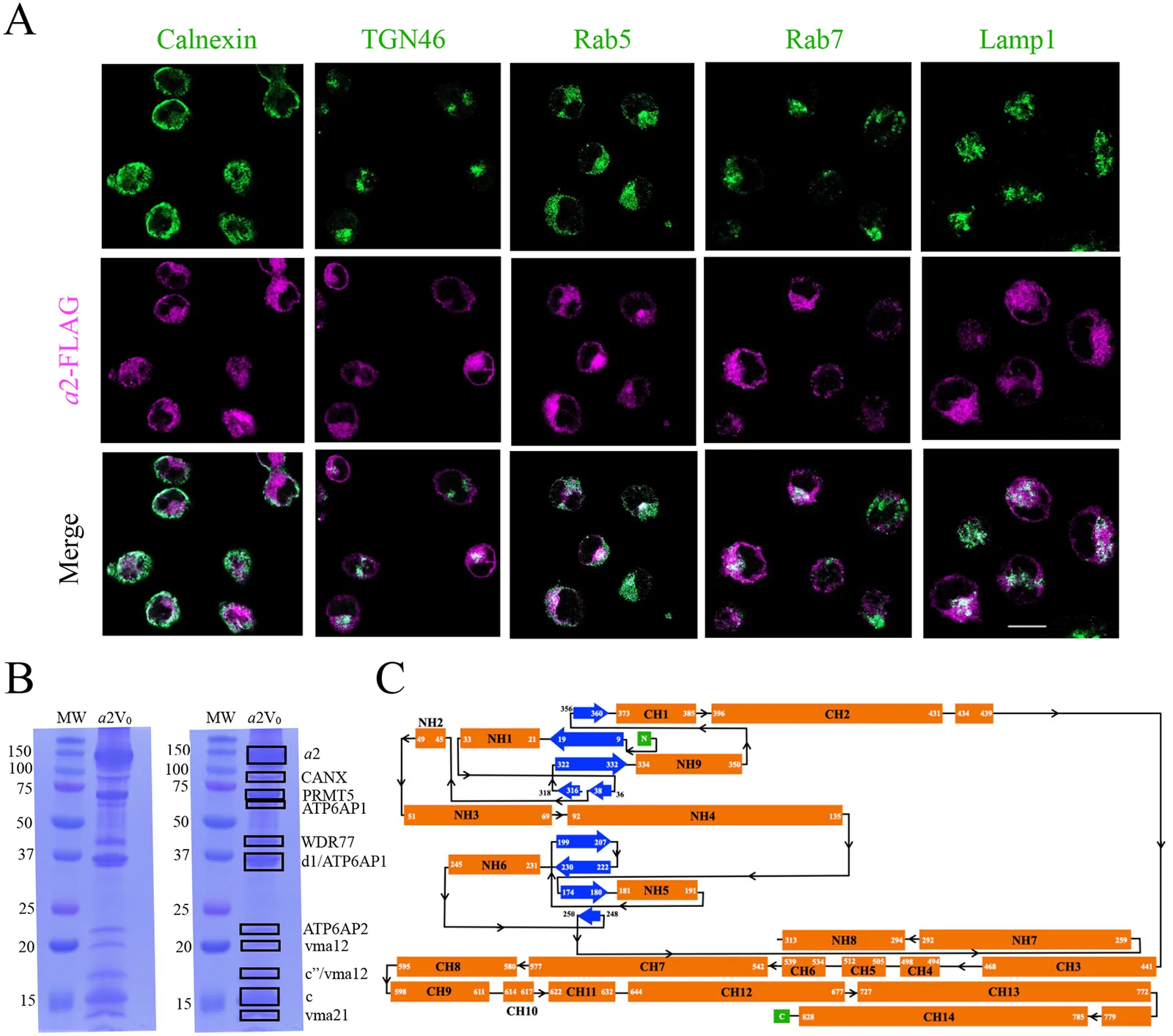
Characterization of *a*2V0. *A*, Immunofluorescence images of *a*2-isoform expressing stable HEK293F cells probed for anti-FLAG antibody (magenta) and antibodies for organelle markers (green). The scale bar represents 10 μm. *B*, Coomassie stained SDS-PAGE of *a*2V_0_ with the right panel showing bands that were cut out for identification with mass spectrometry. Subunits have been labeled based on mass spectrometry data. *C*, Topology of *a*2 based on the *a*2V_0_ structure with helices in orange and β sheets in blue. The topology was generates using PDBsum1^78^.

**Extended data figure 2.**
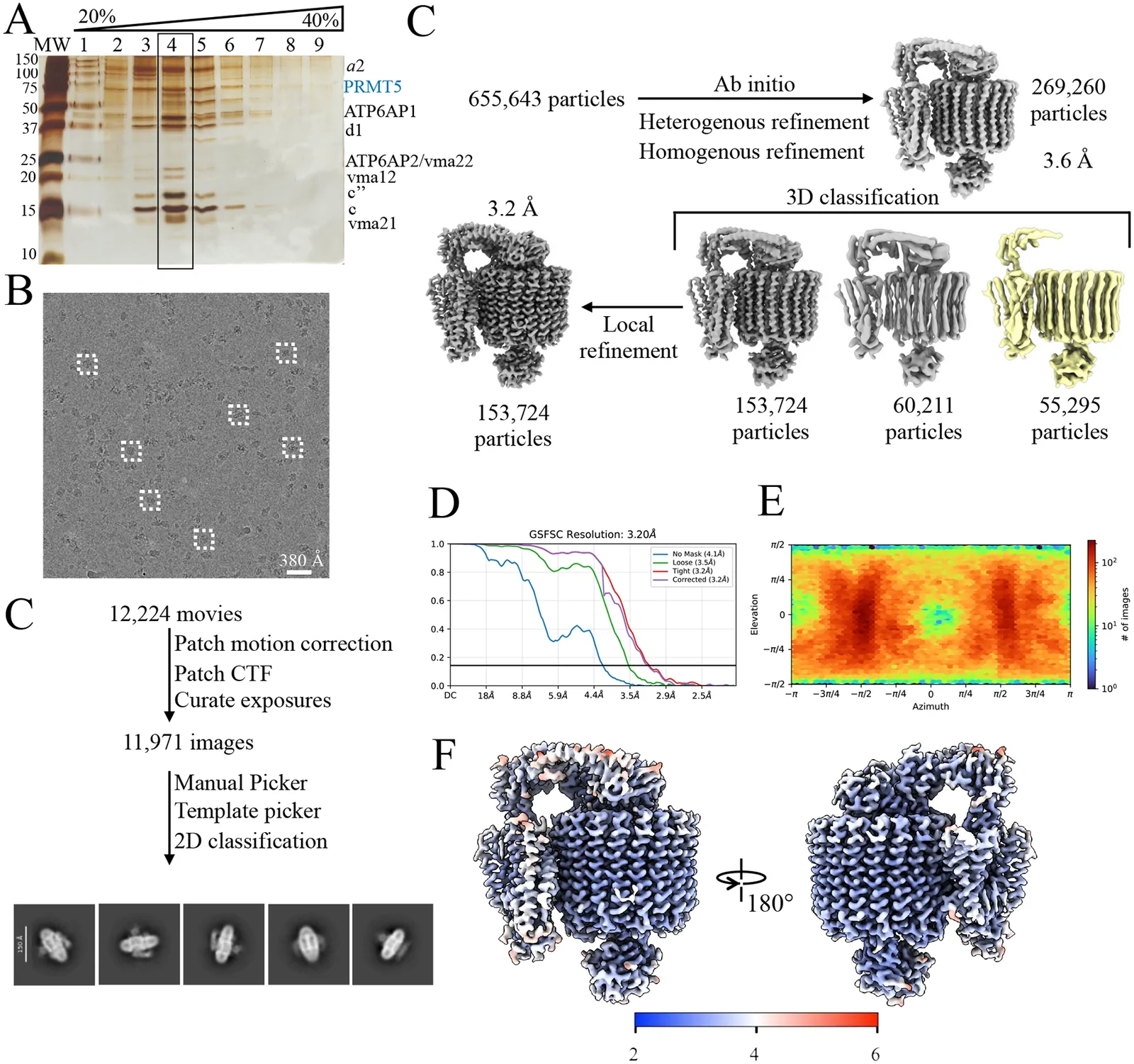
Cryo-EM image processing workflow for *a*2V0. *A*, Silver stained SDS-PAGE of glycerol gradient fractions with the peak fraction highlighted. PRMT5 contaminant is highlighted in blue. *B,* Representative cryo-EM image of *a*2V_0_ with individual particles indicated with white boxes. *C*, Cryo-EM image processing pipeline *D*, Fourier shell correlation (FSC) curves of half-maps. *E*, Particle angular distribution for the final map. *F*, Local resolution estimation of the final map.

**Extended data figure 3.**
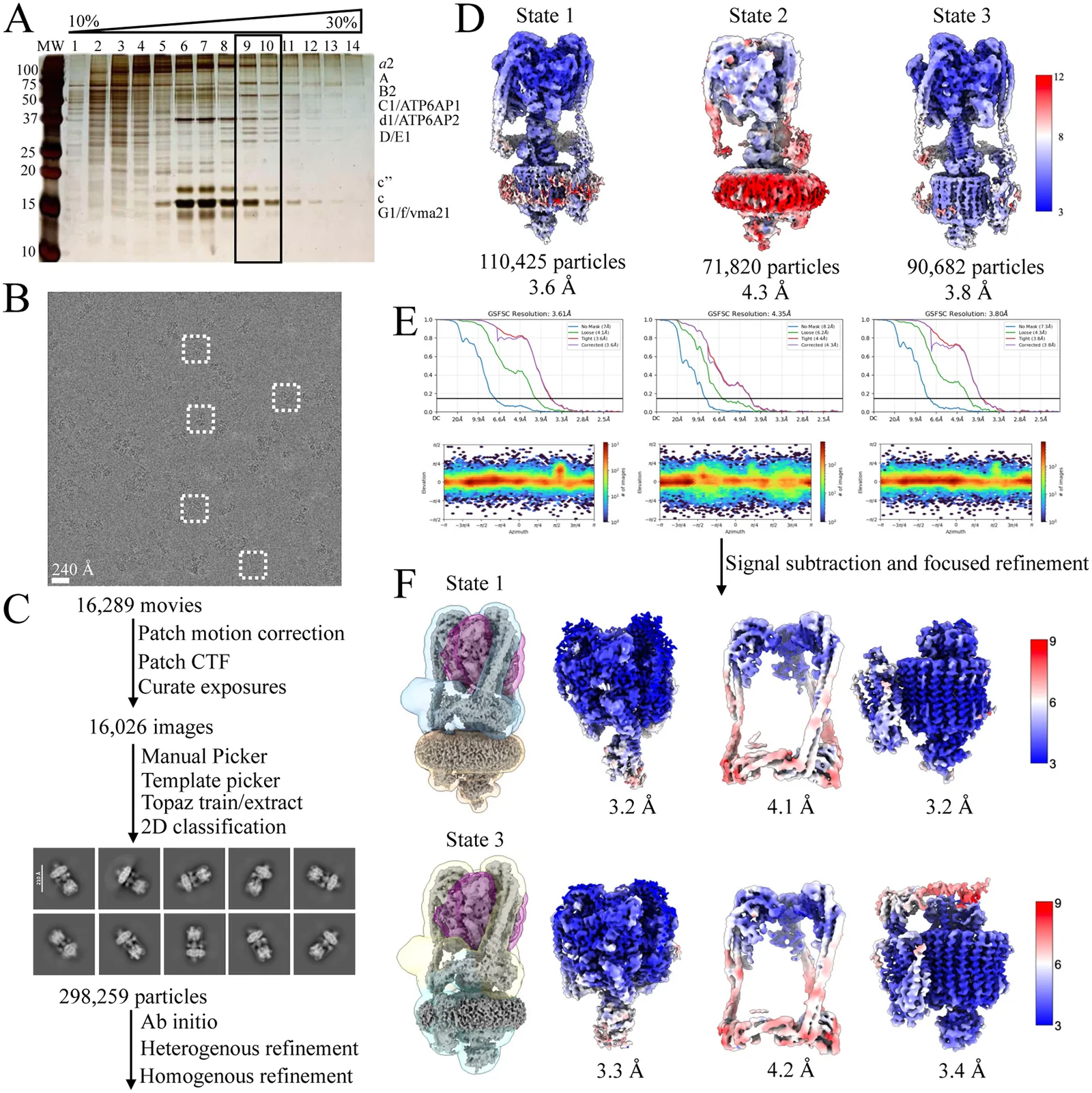
Cryo-EM image processing workflow for *a*2V-ATPase. *A*, Silver stained SDS-PAGE of glycerol gradient fractions with the peak fractions highlighted. *B,* Representative cryo-EM image of *a*2V-ATPase with individual particles indicated with white boxes. *C*, Cryo-EM image processing pipeline *D*, *a*2V-ATPase maps colored according to local resolution, in States 1, 2 and 3. *E*, Fourier shell correlation (FSC) curves (top) and particle angular distribution (bottom) for each State from *D*. *F*, Focused classification of States 1 and 3.

**Extended data figure 4.**
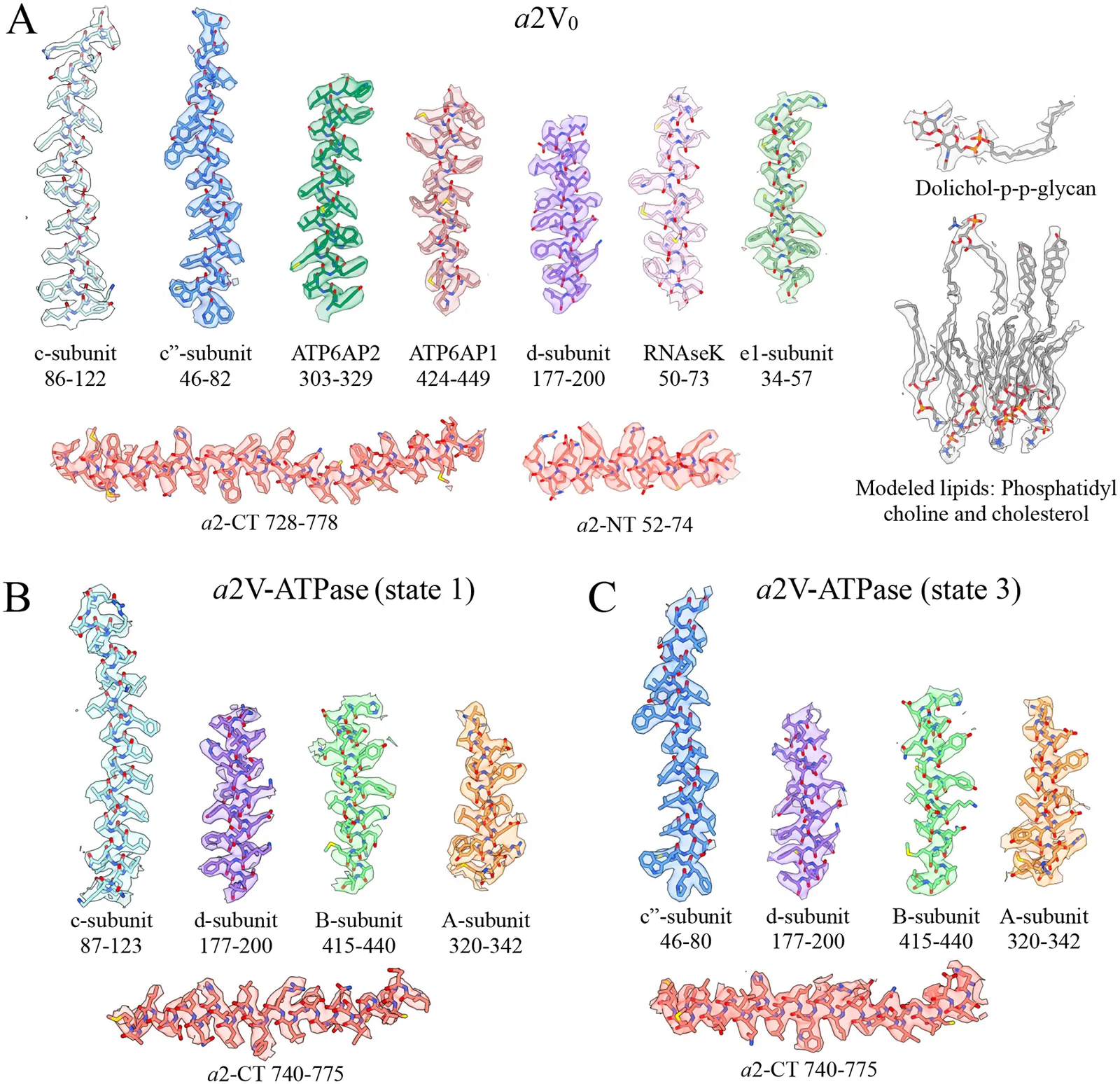
Representative cryo-EM density with the corresponding model. *A*, Cryo-EM density for all subunits of *a*2V_0_ along with modeled lipids. *B* and *C*, Cryo-EM density for selected V_1_ and V_0_ subunits from State 1 (*B*) and State 3 (*C*).

**Extended data figure 5.**
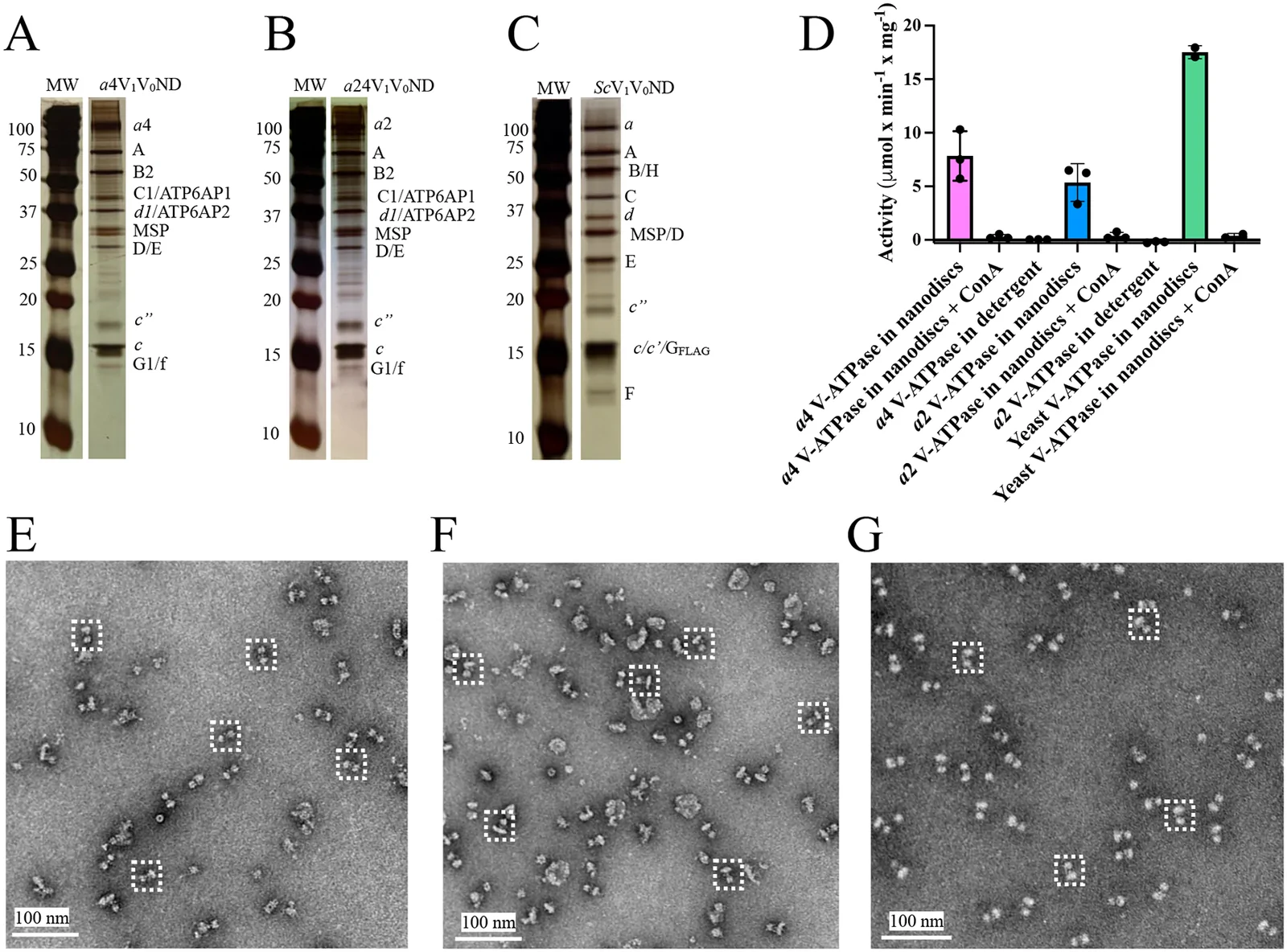
Comparison of *a*2V-ATPase with *a*4V-ATPase and yeast V-ATPase. *A, B* and *C,* Silver stained SDS-PAGE of purified *a*4V-ATPase in lipid nanodiscs, *a*2V-ATPase in lipid nanodiscs and yeast V-ATPase in lipid nanodiscs, respectively. *D*, ATPase activities of V-ATPases conducted using an ATP regenerating assay. *E*, Negative stain electron micrograph of *a*4V-ATPase in lipid nanodiscs. *F*, Negative stain electron micrograph of *a*2V-ATPase in lipid nanodiscs. *G*, Negative stain electron micrograph of yeast V-ATPase in lipid nanodiscs.

**Extended data figure 6.**
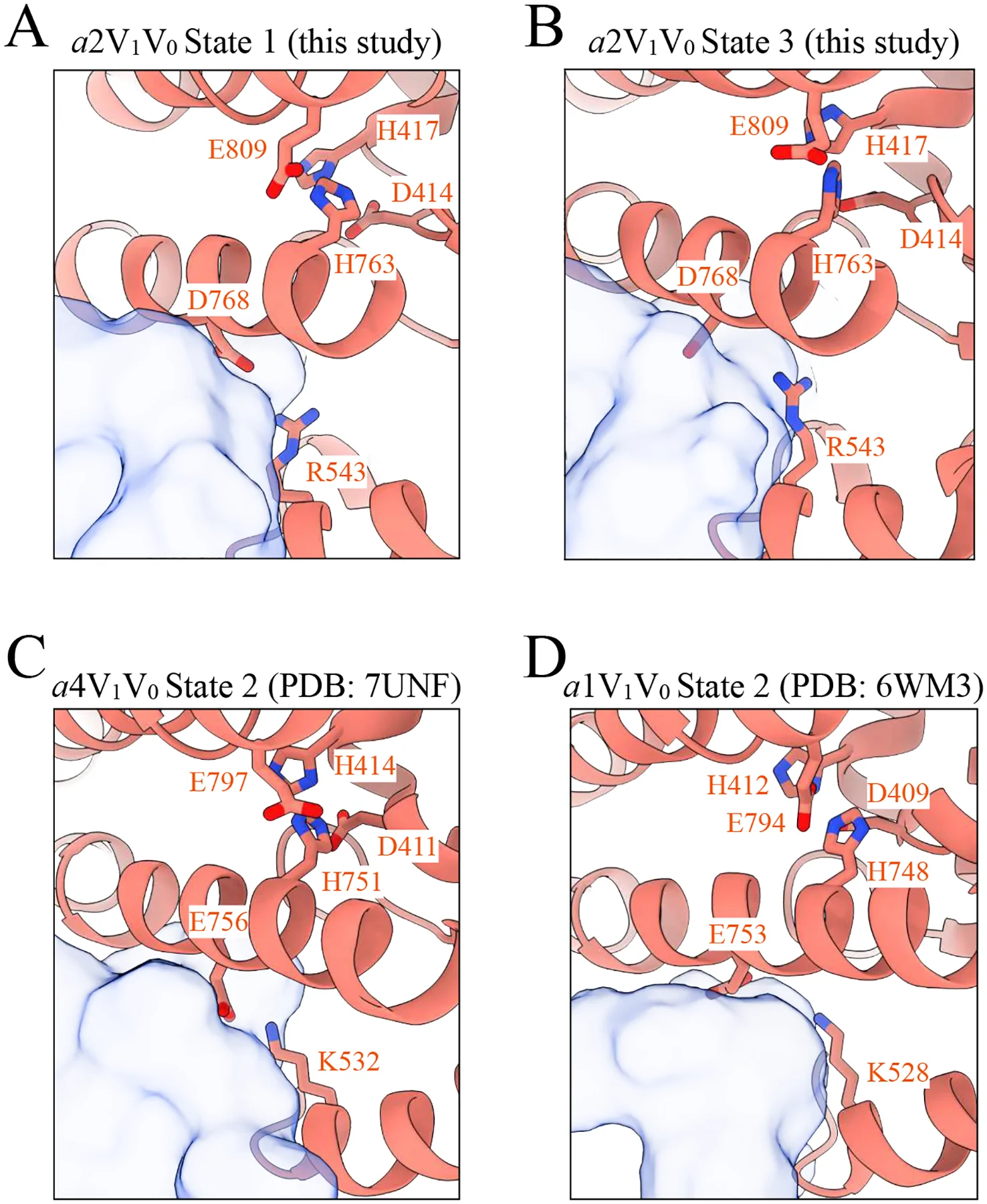
Luminal cavities in assembled V-ATPase. Luminal cavity and surrounding polar residues in *a*2V-ATPase State 1 (*A*), *a*2V-ATPase State 3 (*B*), *a*4V-ATPase State 2 (PDB: 7UNF)^6^ (*C*) and *a*1V-ATPase State 2 (PDB: 6WM3)^5^ (*D*).

**Extended data figure 7.**
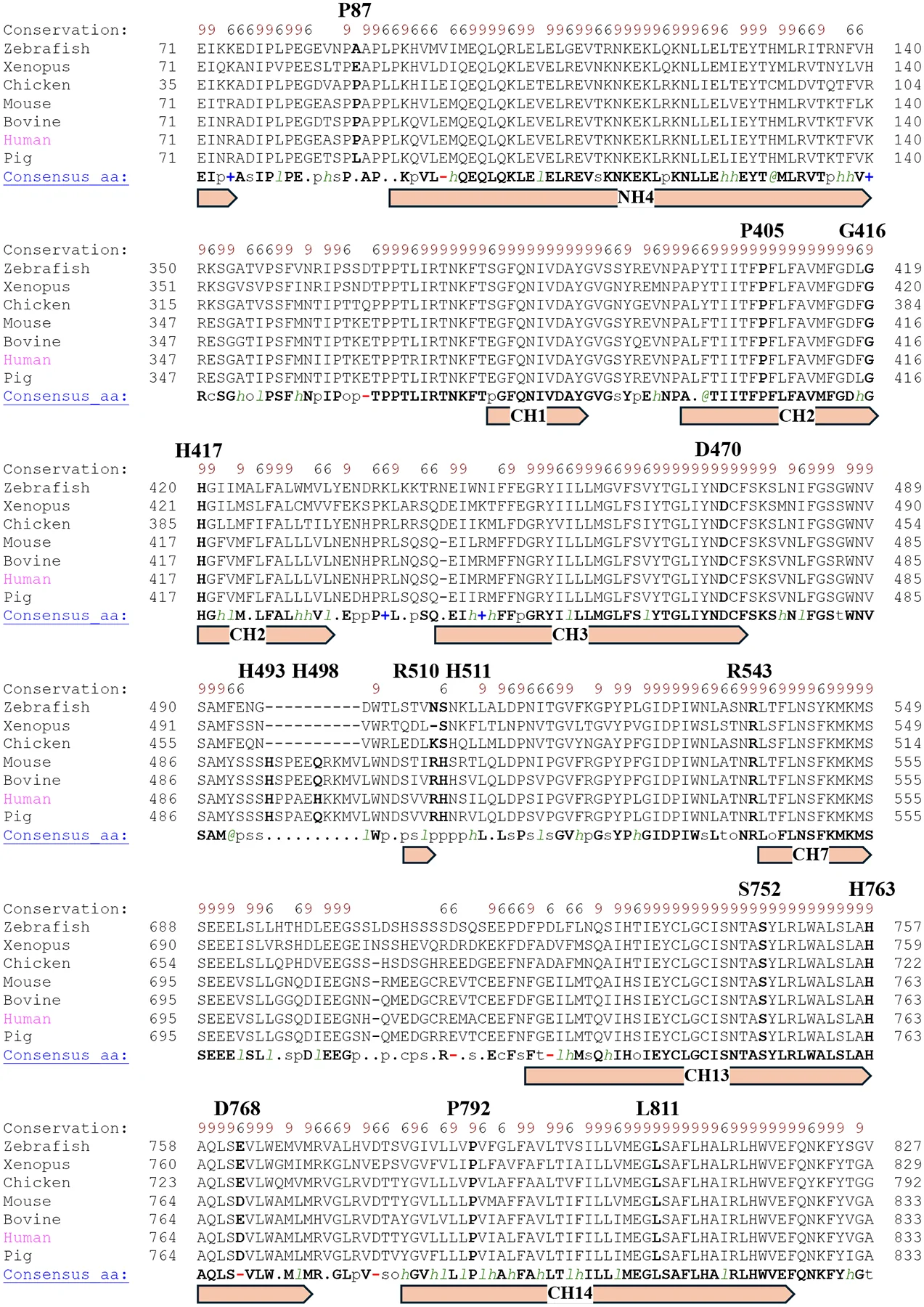
Sequence alignment of *a*2-isoform across vertebrates. Sequence alignment was conducted using PROMALS3D^79^. The first line in each block shows conservation indices for positions with a conservation index above 4 (9 being highly conserved). Consensus amino acids (a.a) symbols are as follows - aliphatic residues (**I, V, L**): *l;* aromatic residues (**Y, H, W, F**): @; hydrophobic residues (W, F, Y, M, L, I, V, A, C, T, H): *h;* alcohol residues (**S, T**): o; polar residues (D, E, H, K, N, Q, R, S, T): p; tiny residues (**A, G, C, S**): t; small residues (A, G, C, S, V, N, D, T, P): s; bulky residues (E, F, I, K, L, M, Q, R, W, Y): b; positively charged residues (K, R, H): +; negatively charged residues (D, E): -; charged (D, E, K, R, H): c.

## Supplementary Material

Supplementary Files

This is a list of supplementary files associated with this preprint. Click to download.

Extendeddatamovie1.mp4

Extendeddatamovie2.mp4

Extendeddatamovie3.mp4

**Extended data movie 1. Luminal extension map and model.** Rocking movie showing the cryo-EM map and model of the luminal extension of *a*2, as observed in the *a*2V_0_ structure.

**Extended data movie 2. Salt bridge that blocks the luminal cavity.** Rocking movie showing the salt-bridge between *a*2R543 and *a*2D768.

**Extended data movie 3. MD simulation of salt bridge.** Snippet of MD simulation showing *a*2R543 switching between *a*2D768 and *a*2D470.

## Figures and Tables

**Figure 1.**
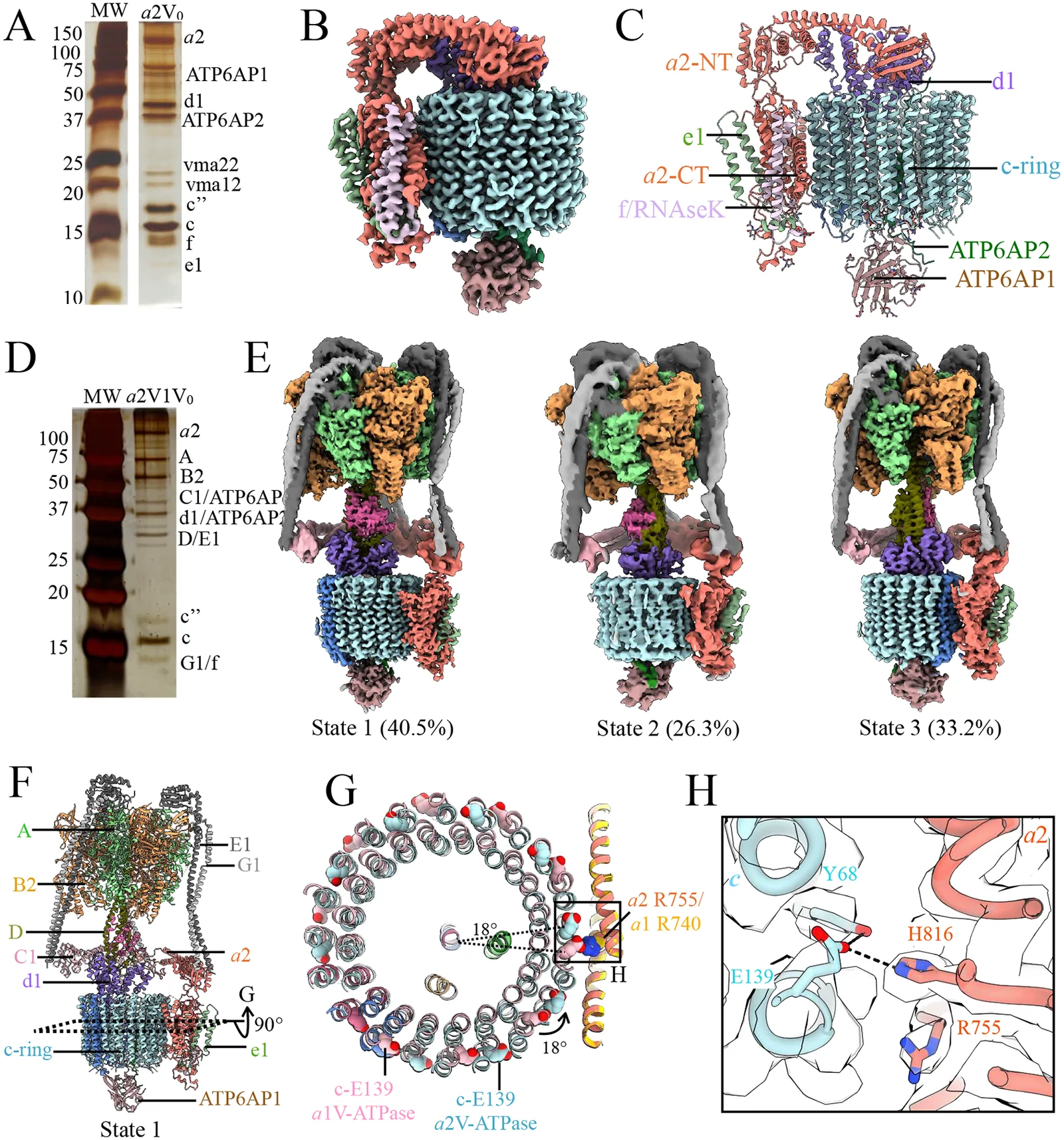
Cryo-EM structure of *a*2V0 and *a*2V-ATPase. *A*, Silver stained SDS-PAGE of purified *a*2V_0_. *B*, Cryo-EM map of *a*2V_0_. *C*, Atomic model of *a*2V_0_. *D*, Silver stained SDS-PAGE of *a*2V-ATPase. *E*, Cryo-EM maps of *a*2V-ATPase in three rotational states. *F*, Atomic model of *a*2V-ATPase in State 1. *G*, Sectional view of V_0_ in *a*2V-ATPase (state 1) overlaid with *a*1V-ATPase (state 1) (PDB: 6WM2)^5^ with the *a*2V-ATPase c-ring in blue and *a*2 in salmon color, and *a*1V-ATPase c-ring in pink and *a*1 in yellow. The c-ring glutamates and the *a*-subunit Arginine are depicted as spheres. *H*, Enlarged view of the highlighted area at the *a*2/c interface in *G* is shown with the corresponding cryo-EM density. Distance between cE139 and *a*2H816 is ~3.6 Å and between cE139 and cY68 is ~3.4 Å.

**Figure 2.**
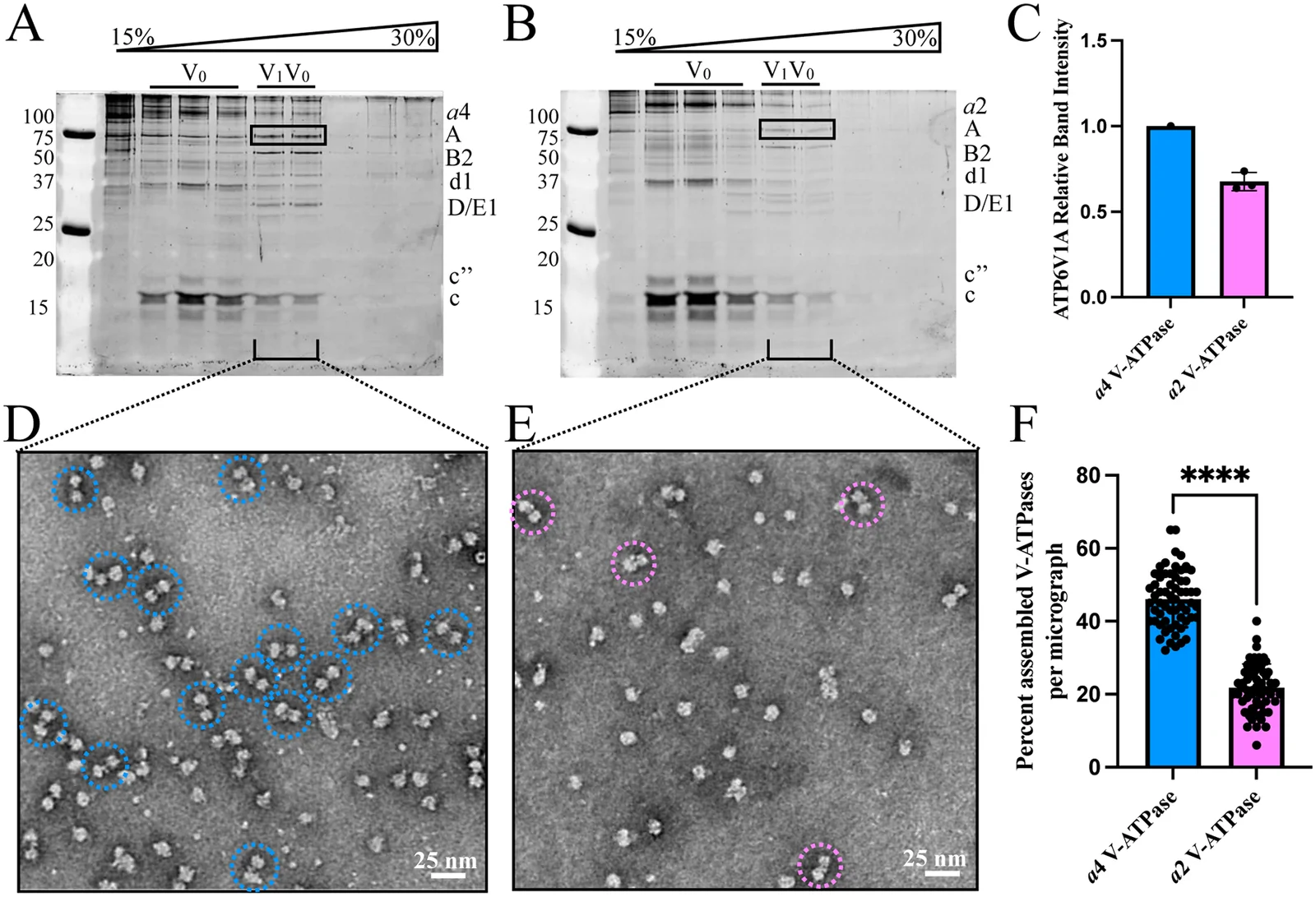
Stability of *a*2V-ATPase versus *a*4V-ATPase. SYPRO Ruby stained SDS-PAGE of glycerol gradient fractions from *a*4V-ATPase (*A*) and *a*2V-ATPase (*B*). *C*, Quantification of the band intensity for V_1_A (highlighted in *A* and *B*). Mean ± standard deviation from three separate purifications is shown. The V_1_V_0_ fractions highlighted in *A* and *B* were used for negative stain electron microscopy. Representative micrographs are shown for *a*4V-ATPase (*D*) and *a*2V-ATPase (*E*). *F*, Quantification of the percentage of assembled *a*4V-ATPase versus *a*2V-ATPase. Each data point represents one micrograph, and 20 micrographs were analyzed per purification of *a*4V-ATPase and *a*2V-ATPase, for three separate purifications of each. Statistical significance between the two groups was determined using an unpaired, two-tailed Welch’s t-test with a value of <0.05 considered significant.

**Figure 3.**
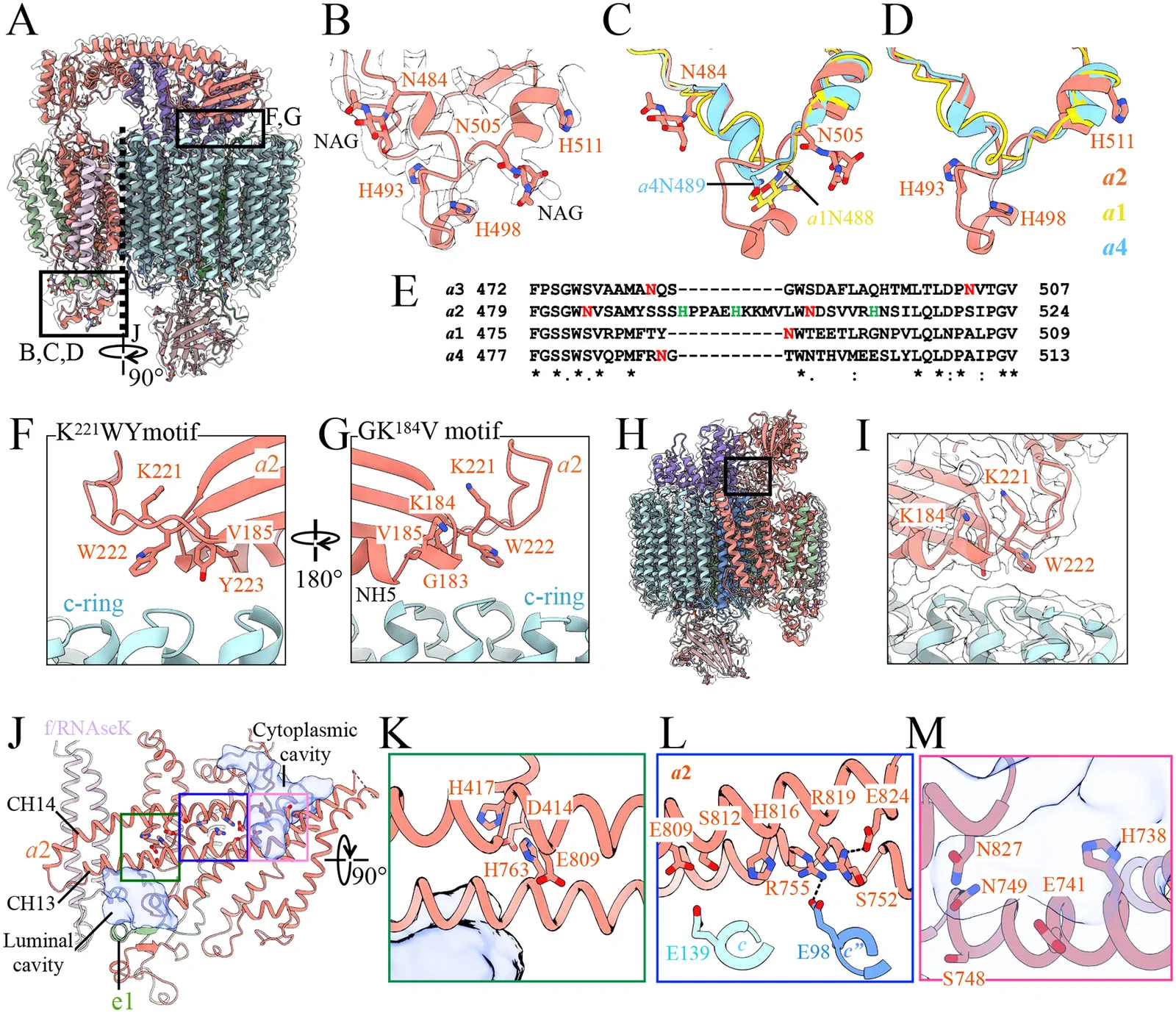
Distinguishing structural features in *a*2V0. *A,* Atomic model of *a*2V_0_ fit into its cryo-EM density. *B*, Luminal extension of *a*2V_0_ as highlighted in *A* with glycosylation sites and non-conserved histidines depicted in stick. *C* and *D,* Overlay of the luminal extension from *a*1 (PDB: 6WM4)^5^, *a*2 (this study) and *a*4 (PDB: 7UNF)^6^ structures. The glycosylation sites are compared in *C*, while the position of histidines is compared in *D*. *E*, Sequence alignment of human *a*-isoforms for the region depicted in *B, C* and *D*. *F* and *G*, Membrane facing β hairpin loops in the distal region of *a*2NT highlighted in *A*, with *F* showing the K^221^WY motif and *G* showing the GK^184^V motif. *H*, Map and model of *a*2V_0_ rotated 120° counterclockwise with respect to *A*. *I*, Enlargement of the highlighted region in *H* showing cryo-EM density for K221 and K184. *J*, 90° rotated slice of *a*2V_0_ *as* depicted by dashed lines in *A,* showing the interface of *a*2CT that interacts with the c-ring. *K, L* and *M* are enlarged regions from *J* as depicted by color coded boxes.

**Figure 4.**
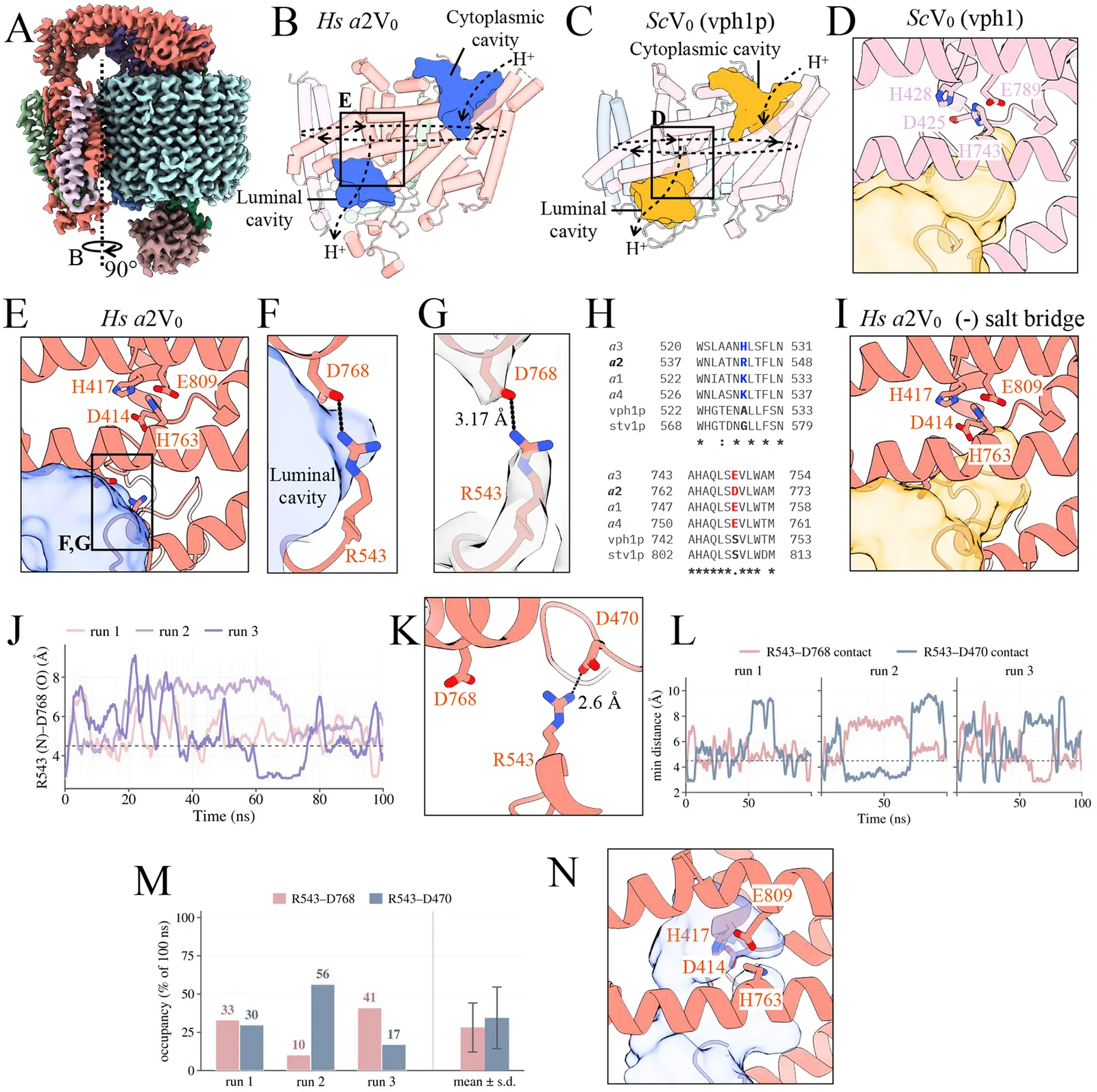
A salt bridge limits access of the luminal cavity. *A*, Cryo-EM map of *a*2V_0_. *B*, 90° rotated slice of *a*2V_0_ as depicted by dashed lines in *A,* revealing the interface of *a*2CT that interacts with the c-ring. Cytoplasmic and luminal cavities are depicted in blue. Dotted lines with arrows represent the direction of proton flow. *C*, A similar depiction of *a*CT from the structure of yeast V_0_, with the two half-channels colored in yellow. Dotted lines with arrows depict direction of proton flow. *D*, Enlarged view of highlighted region in *C*. *E*, Enlarged view of highlighted region in *B*. *F* and *G*, Enlarged view of highlighted region in *E*, showing a salt bridge between R543 and D768. Corresponding cryo-EM density is shown in *G*. *H*, Sequence alignment of human *a-*isoforms and the two yeast isoforms. *I*, Enlargement of highlighted area in *B*, wherein the luminal cavity is calculated after two replacements - R543A and D768S. *J*, Time evolution of the distance between the R543 (N) and D768 (O) atoms across three independent 100 ns MD runs, illustrating the intermittent formation and breaking of the salt bridge. The horizontal dashed line indicates the 4.5 Å cutoff. *K*, Representative structural snapshot of an alternative conformation observed during MD simulation wherein a salt bridge is observed between R543 and D470. *L*, Time evolution of the salt bridge distance for R543–D768 (pink) and R543–D470 (blue) across three independent 100 ns MD simulation runs. The horizontal dashed line represents the 4.5 Å cutoff used for defining salt bridge formation. *M*, Percentage occupancy of the R543–D768 and R543–D470 salt bridges across individual simulation runs and their overall average, calculated based on the fraction of time the distance remained below the 4.5 Å threshold. *N*, Luminal cavity calculated using coordinates of the structural snapshot shown in *K* with the R543–D470 salt bridge.

**Figure 5.**
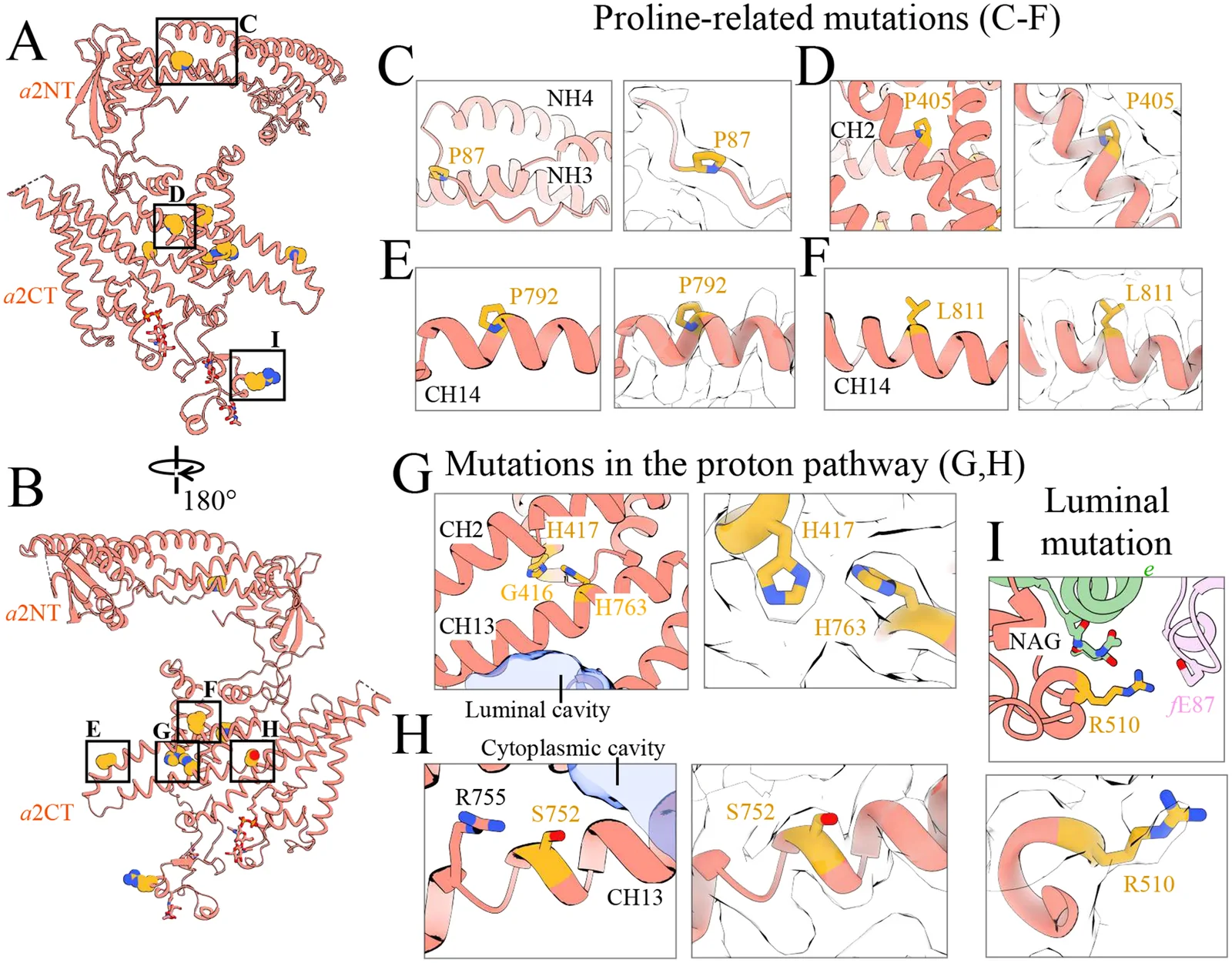
Mapping of missense mutations in the *a*2-isoform that cause cutis laxa. *A* and *B*, Atomic model of *a*2 with the locations for missense mutations depicted as yellow spheres and labeled in yellow. *C, D, E* and *F,* Enlarged highlighted regions in *A* and *B* that represent proline-related mutations. The model for each panel is shown on the left with the corresponding cryo-EM map on the right. *G* and *H*, Enlarged highlighted regions in *B* that represent mutations in the proton pathway. The model for each panel is shown on the left with the corresponding cryo-EM map on the right. *I*, Enlarged highlighted region in *B* that represents a single reported mutation in the luminal extension. The model is shown on top with the corresponding cryo-EM map on the bottom.

**Table 1.** Cryo-EM data collection, refinement and validation statistics

	1. *a*2V0	2. *a*2V-ATPase State 1	3. *a*2V-ATPase State 2	4. *a*2V-ATPase State 3
EMDB	EMD-79136	V_1_V_0_: EMD-79158 V_0_: EMD-79159 V_1_: EMD-79160 PS: EMD-79161	V_1_V_0_: EMD-79147 V_0_: EMD-79148 V_1_: EMD-79149	V_1_V_0_: EMD-79138 V_0_: EMD-79142 V_1_: EMD-79146 PS: EMD-79145
PDB	38TF	38UA		38TJ
**Data collection and processing**				
Magnification	92,000x	92,000x	92,000x	92,000x
Voltage (kV)	200	200	200	200
Electron exposure (e^−^/Å^2^)	40	40	40	40
Defocus range (μm)	−0.8 to −1.5	−0.8 to −1.4	−0.8 to −1.4	−0.8 to −1.4
Pixel size (Å)	1.1	1.1	1.1	1.1
Symmetry imposed	C1	C1	C1	C1
Initial particle images	2,506,166	1,425,496	1,425,496	1,425,496
Final particle images	153,724	110,425	71,820	90,682
Map resolution (Å)	3.2	V_1_V_0_: 3.6 V_0_: 3.2 V_1_: 3.2 PS: 4.1	V_1_V_0_: 4.3 V_0_: 4.1 V_1_: 3.4	V_1_V_0_: 3.8 V_0_: 3.4 V_1_: 3.3 PS: 4.2
	
	
	
**Refinement**				
Initial model used (PDB ID)	6WM4	6WM2		6WM4
Model resolution at FSC=0.5 (Å)	3.5	4.3		4.5
Map sharpening B factor (Å^2^)	−72.2	Locally sharpened		Locally sharpened
Model composition				
Non-hydrogen atoms	24198	58068		57716
Protein residues	3082	7882		7922
Ligands	24	1		1
B factors (Å^2^)				
Protein residues	71.87	154.13		181.1
Ligands	83.28	154.17		192.6
R.M.S deviations				
Bond lengths (Å^2^)	0.003	0.005		0.005
Bond angles (°)	0.814	1.12		1.13
Validation				
MolProbity score	1.24	2.04		2.22
Clashscore	4.64	16.12		15.66
Poor rotamers (%)	0.53	0.93		1.57
Ramachandran plot				
Favored (%)	98.16	95.25		94.57
Allowed (%)	1.77	4.6		5.26
Disallowed (%)	0.07	0.14		0.17

## Data Availability

3D cryo-EM density maps of human V-ATPase with the *a*2-isoform have been deposited in the Electron Microscopy Data Bank as follows – *a*2V_0_ (EMD-79136), *a*2V-ATPase in state 1 (EMD-79158, EMD-79159, EMD-79160, EMD-79161), *a*2V-ATPase in state 2 (EMD-79147, EMD-79148, EMD-79149), and *a*2V-ATPase in state 3 (EMD-79138, EMD-79142, EMD-79146, EMD-79145). The corresponding coordinates for atomic models have been deposited in the Protein Data Bank as follows – *a*2V_0_ (38TF), *a*2V-ATPase in state 1 (38UA), and *a*2V-ATPase in state 3 (38TJ) (Table 1).
